# Perspectives before incremental trans-disciplinary cross-validation of clinical self-evaluation tools and functional MRI in psychiatry: 10 years later

**DOI:** 10.3389/fpsyt.2022.999680

**Published:** 2022-10-10

**Authors:** Drozdstoy Stoyanov

**Affiliations:** Department of Psychiatry and Medical Psychology and Research Institute, Plovdiv Medical University, Plovdiv, Bulgaria

**Keywords:** diagnosis, translation, validity, functional neuroimaging, clinical assessment and patient diagnosis

## Abstract

Translational validity (or trans-disciplinary validity) is defined as one possible approach to achieving incremental validity by combining simultaneous clinical state-dependent measures and functional MRI data acquisition. It is designed under the assumption that the simultaneous administration of the two methods may produce a dataset with enhanced synchronization and concordance. Translational validation aims at “bridging” the explanatory gap by implementing validated psychometric tools clinically in the experimental settings of fMRI and then translating them back to clinical utility. Our studies may have identified common diagnostic task-specific denominators in terms of activations and network modulation. However, those common denominators need further investigation to determine whether they signify disease or syndrome-specific features (signatures), which, at the end of the day, raises one more question about the poverty of current conventional psychiatric classification criteria. We propose herewith a novel algorithm for translational validation based on our explorative findings. The algorithm itself includes pre-selection of a test based on its psychometric characteristics, adaptation to the functional MRI paradigm, exploration of the underpinning whole brain neural correlates in healthy controls as compared to a patient population with certain diagnoses, and finally, investigation of the differences between two or more diagnostic classes.

## Introduction

The issue of validity and validation in psychiatry (2013, 2014, and 2015).

The validity of psychiatric diagnosis and nosology was considered a major source of controversy in the past decade (1–4). The critical approaches to the nosological structure of psychiatry are grounded in epistemological concerns about its conventional origins, whereas most other medical disciplines usually triangulate diagnostic constructs upon molecular, pathophysiological, and imaging biomarkers to co-produce robust criteria to sustain diagnosis at the nosological level (5). The continuous failure of neuroscience and neurobiology to deliver such fingerprints of disease, relatively independent from clinical observations, is seen as one of the possible limitations of the existing classification systems (6). It becomes evident that those nosological systems are highly inconsistent regardless of the number of revisions since 1989 (7). This is further complemented by the replication crisis in the field of functional MRI, both when it comes to normal populations and patient samples. In addition, as a major caveat, it is assumed to be the mono-disciplinary structure of the different evaluation methods emerging in two discrepant fields of knowledge. In other words, research studies are either performed by means of stringent functional MRI experimental tasks or by means of psychometric assessment rating scales separately (8). However, those two groups of methods are essentially divergent, separated by the so-called explanatory gap. They belong to two discrete disciplinary and nomothetic systems, which have limited resources for translation from one to the other. There are minimal attempts to unite them into the same toolkit, as discussed in the next sections.

### Trait (state-independent) measures: A case study on TCI-r

State-independent (trait) measures are widespread in psychopathology, especially in the definition and study of the mechanisms underlying endophenotypes. They are convenient, as long as the test-retest stability of the construct measures is established, and are usually performed in independent time slots from neuroimaging studies. Various correlations of such dimensions have been reported, e.g., harm avoidance and self-directedness with fMRI and magnetic resonance spectroscopy (MRS) (9–11). However, the visual stimuli employed in functional MRI are not derived from the inventory itself but represent visualizations designed for the imaging data acquisition supposedly related indirectly to the trait construct in question.

### State-dependent measures: What is clinically relevant?

Despite the advances in the construction of endophenotypes, most of the clinical conditions in psychiatry are state-dependent, with many confounds pertinent to time-of-the-day dynamics, especially for affective disorders (3). This factor has also been acknowledged as a general concern in functional neuroimaging. Hemodynamic response function in the resting state is also influenced by the time-of-the-day fluctuations of the BOLD signal (see Vaisvilaite et al. (12) for more details). Those are certainly even more implicated as a potential caveat in task-related measures. Therefore, one underestimated challenge would be better synchronizing the state-dependent tests with functional MRI data acquisition (13). Nevertheless, the entire field seems to overlook the state-dependent tests as more difficult to adapt and interpret in fMRI paradigms and to provide retest stability to ensure robust replications.

### Incremental validity

Another unresolved issue remains incremental validity in psychometric studies (2). There are many conceptual concerns raised about the conventional definitions of validity in mental health disciplines (14). Incremental validity is usually achieved in two ways. Increasing the number of scale items or increasing the Likert scale from 5 to 7 degrees would be examples of intra-correlation. However, it is intra-correlative as long as the reported correlations are measured within the same disciplinary domain, here, the psychometrics domain.

The other way to address incremental validity is by adding external inter-correlative tests, which consistently explore independent indexes from other disciplinary domains (such as functional neuroimaging or sociological measures, for instance), which converge upon a given psychometric construct. On the other hand, neuroimaging studies tend to establish validity in a hypothesis-driven fashion; this suggests that *a priori* hypothesis about implicated functional brain regions of interest (ROI) (modules, networks) and their modulation are entered as variables into a model to be tested under experimental conditions. More or less naturalistic stimuli (proximal to everyday environmental demand and clinical assessment methods) and contextual factors are ignored to grant control of the experimental settings. This experimental control narrows the validity of the implemented fMRI tasks and produces decontextualized, rigid data with poor replicability and generalizability outside the specific experimental environment. This is a prototype of provisionally speaking, “decremental” validity—the opposite of incremental—which delivers “sterile” results with limited possibilities to translate research findings into clinical practice.

Both contemporary neuroimaging and psychopathology aim at incremental validity within their own disciplinary domains, and any potential cross-validation measures are estimated *post-hoc*.

### Translational validity and fMRI

Translational validity (or trans-disciplinary validity) is defined as one possible approach to incremental validity combining simultaneous clinical state-dependent measures and functional MRI data acquisition (3, 15). It is designed under the assumption that the simultaneous administration of the two methods may produce a dataset with enhanced synchronization and concordance (4). Translational validation aims at “bridging” the explanatory gap by implementing validated psychometric tools clinically in the experimental settings of fMRI and then translating them back to clinical utility. This is required to deliver the meta-language of psychiatry, which may combine and transcend the diverse disciplinary languages involved in psychiatry (3). The sections below outline the contributions of different groups supporting this concept.

## Advances in the field from 2010–2020

### Studies with cognitive tests in healthy populations: Raven test

Progressive matrices, non-verbal intelligence tests by Raven, were among the first psychometric tools implemented for functional MRI in 2001 (16). More precisely, selected progressive matrices have been adapted as stimuli to yield activations in the dorsolateral prefrontal cortex during reasoning. Without explicit aim at validating Raven's test with fMRI, the authors have discovered by implication specific neuro-correlates that underpin relational complexity (2-relational vs. 1-relational) problems of the Raven matrices. Unfortunately, the sample size was too small (a total of ten subjects) to draw any robust inference from that study. Further studies (17) also reported using Raven's test to assess the involvement of the postero-lateral prefrontal cortex in relational processing from a developmental perspective.

### Studies with other cognitive tests in normal populations and disease-specific applications

One successful adaptation of clinically relevant tests to the context of fMRI is the application of the paced auditory serial addition test, initially in normal samples and then in a sample of patients with neurological disorders (18). The test was initially set for auditory stimuli and then adapted to visual for the functional MRI paradigm. Activations include the left prefrontal cortex, bilateral cingulate gyrus, left inferior parietal lobule, among others (in healthy controls), and left supra-marginal gyrus in patients with remitting multiple sclerosis and a certain level of cognitive decline (19, 20). Besides, Forn and his associates also implemented the widely used Symbol-Digit-Modalities test (SDMT), with frontal and parietal areas involved in the performance (21). The same test has many implications for clinical practice, especially in evaluating cognitive deficits in neurodegenerative disorders and multiple sclerosis (22). The Sternberg memory test (23) is also compatible with functional MRI settings.

### Studies with affective tests: The Rorschach test and complex social-affective tests

Of the affective tests (a.k.a. projective methods), one of the most commonly used in clinical and expert evaluation is the inkblot test of Rorschach. It has also been adapted to the functional MRI paradigm (24–26), with the more explicit goal of revealing the BOLD signal underlying the psychological test responses. Various task-specific regions are reported outside the visual processing and dorsal attention network systems, which are apparently task-non-specific. Interestingly, sub-cortical areas of the limbic system are involved. Compared with neutral pictures, pictures from the thematic-apperception test have also been adapted for functional MRI since 2006, especially in the study of personality disorders (27).

### Studies with the word association test by CG Jung

Most interesting studies have emerged since 2013 with attempts to determine the functional MRI patterns behind Carl Gustav Jung's Word Association Test. In the 2018 study by Escalimilla and associates (28), the authors recorded two subsequent sessions with WAT on video. They identified the complex-triggering words on the individual level by evaluating verbal and non-verbal behavior and indexing certain reactions as relevant or signifying unconscious relevance to complexes. Then, the same words were presented during the fMRI session in active blocks, alternating with supposedly neutral blocks from the same word set. Authors have applied the group independent component analysis for the fMRI toolbox and identified two brain circuits that correlate with the “complex” words. Those were named “memory, body, and action (circuit 1) and memory, emotion, language, and meaning (circuit 2) and are activated during the processing of complex-triggering words. The authors followed a predominantly explorative approach without a specific preliminary hypothesis on the regions of interest (ROIs) to set boundaries for their analysis. In this way, they, in fact, followed and managed to replicate earlier studies on WAT by Petckovsky (29, 30).

## Our contributions, 2017–2022

### Depression scale

Our group has studied the blood-oxygenation-level-dependent (BOLD) signal associated with simultaneous administration of the depression scale by Von Zerssen (31, 32). The scale was selected for various clinical and experimental reasons. The clinical arguments are that it captures in a clear, concise, and condensed form the depressive self-reported symptoms, as opposed to the paranoid syndrome, two of the most common clinical states in clinical practice. The experimental rationale was that the items had a relatively low cognitive load (as compared to the Beck Depression Inventory, for instance) and were, therefore, more likely to correspond to the “phenomenological core” of depression rather than one of its dimensions, namely the cognitive impairment. The items from the scale were constructed in four blocks and alternated against blocks of diagnostically neutral items, thus constituting an interest scale. Those were administered to two groups: patients with a major depressive episode and healthy controls. The results from direct comparison yielded significant residual activation clusters in the middle frontal gyrus after family-wise error correction. This result may indicate that the high depression score in terms of self-evaluation corresponds to a specific pattern of brain activation, which is also related to dysfunctional connectivity of the same region at rest (32, 33).

### Paranoid-depression scale *(PD-S)*

In order to further outline the core depressive symptoms from other common psychopathological conditions, we have expanded the paradigm by including the paranoid items from the paranoid-depressive scale by von Zerssen (34–36). The selection of the scale was based on similar assumptions as described above. It was then administered simultaneously with a functional MRI session to two groups of patients: depression and schizophrenia groups. The results demonstrated significant residual activations in the precuneus and angular gyrus areas, which were considered diagnostically relevant (37). Those activations are considered to be in the default mode network, which means that there is essentially an aberrant activation in that system in schizophrenia (32, 33, 38). In such a way, we have attempted to integrate the two methods: the clinical self-assessment scale and functional MRI, with the intention of achieving incremental trans-disciplinary validity of both methods.

### Multivariate analysis (MLM) of *PD-S;* structural and resting-state functional MRI

As a next step, we have a machine learning methodology to investigate brain signatures behind item responses on different scales (34–36). There were discovered with the multivariate linear method (MLM) in the same data set whole brain signatures behind the item responses on the scales, identified as “clinical loadings.” The positive load of paranoid and neutral items and negative on depression items were correlated with a brain signature, which was able to predict the clinical diagnosis up to 93% (area under the curve).

### IAPS and MLM on IAPS

In other studies of our group, we have identified task-specific activation patterns in the right middle temporal gyrus in patients during the processing of positive compared to neutral images from the International Affective Pictures System (IAPS) (39, 40). Brain signatures were identified using MLM, which can predict with high accuracy the diagnosis. From a certain perspective, IAPS may be considered as a proxy task for projective tests, such as TAT, which is capable of evaluating affectivity in mental disorders (41).

### Stroop n-back test

Our group has recently investigated the functional MRI underpinnings of the Stroop n-back color and word test (SCWT) in depression (42). It is designed to capture basic components of attention by measuring the subject's performance while processing congruent and non-congruent color-word stimuli. Attentional deficits are among the common psychopathological features of depression. On a direct comparison of depressed patients with control subjects, there have been reported deactivations of the lingual and fusiform gyrus in patients as the complexity of the task is increased on color versus word conditions.

**Figure 1:**
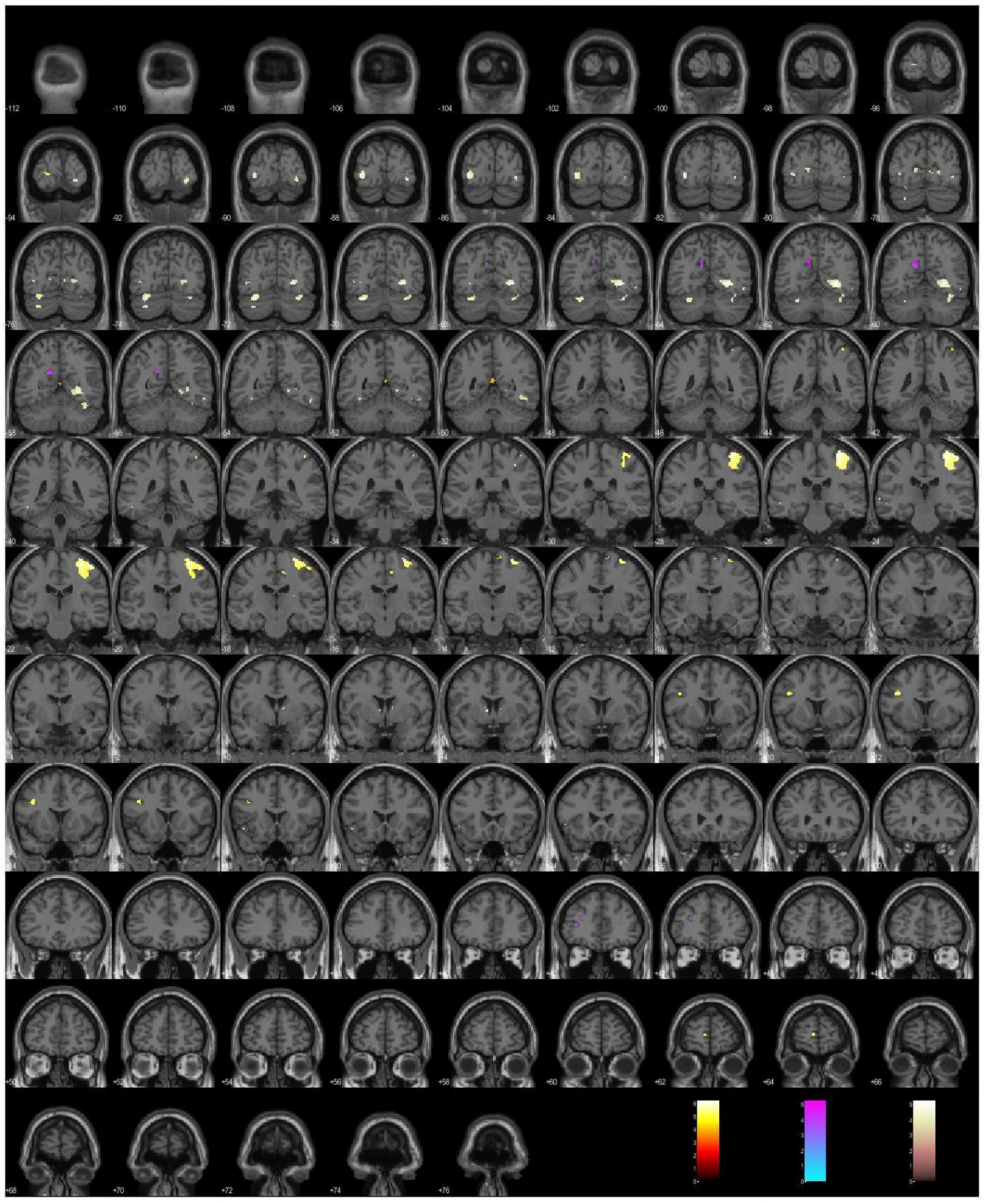
A plot of the differential activations under the simultaneous administration of psychometric tools: the depressive Scale (hot), the paranoid Scale (cool), the Stroop Color, and the Word Test (pink).

To summarize our findings in 2008–2022, we have implemented three major groups of clinical evaluation tools in the context of functional neuroimaging: self-evaluation scales, a proxy for projective testing, and a cognitive assessment test. Each of those psychometric tools has produced differential activations or brain signatures, which are illustrated in Figure 1.

**Figure 2:**
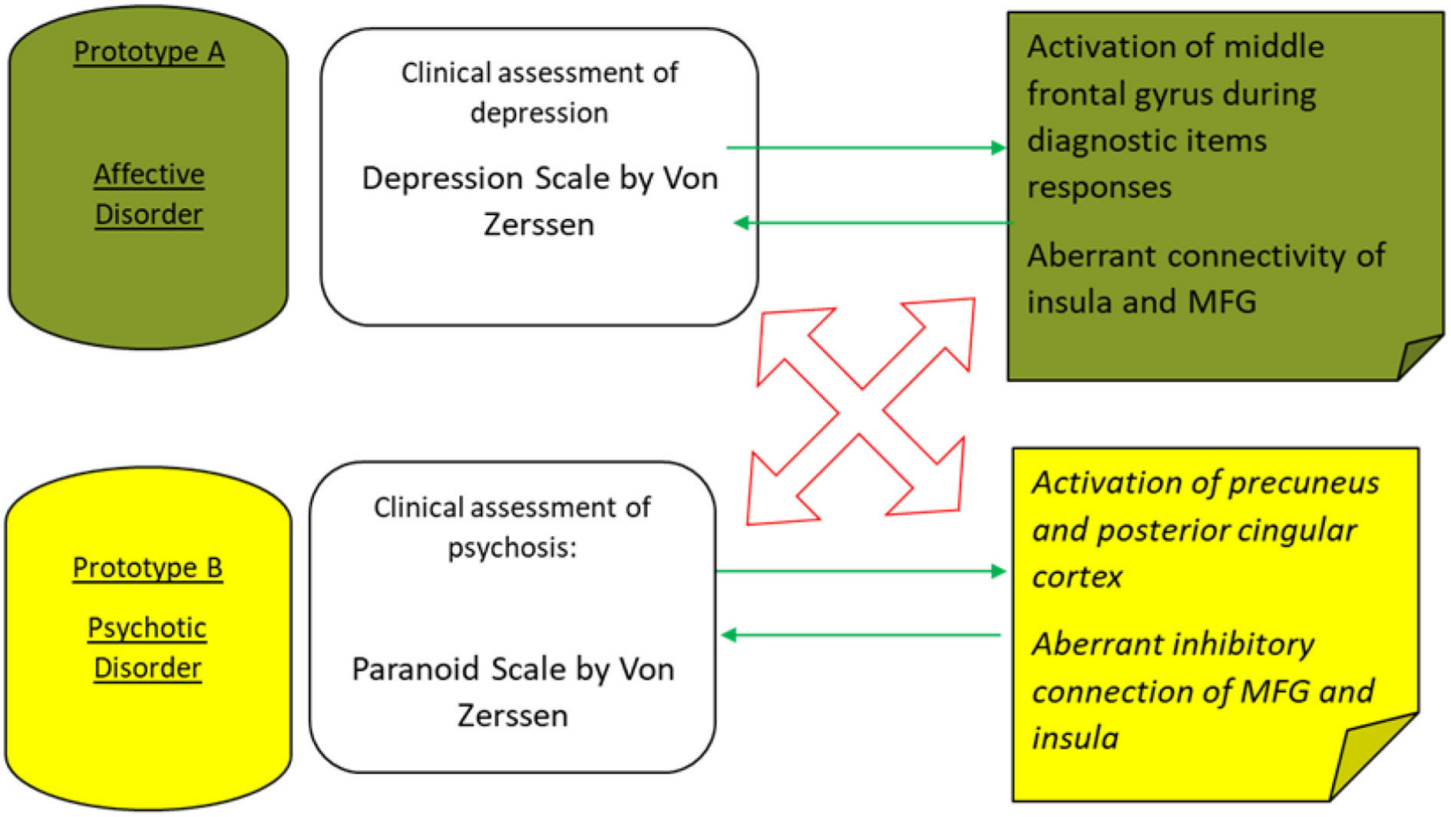
The main convergent validity (green arrows) and divergent validity (red arrows) are outlined to discriminate on an incremental level between two major prototypes of mental disorders—major depressive episodes and schizophrenic psychosis.

In turn, those patterns of brain activations and connectivity aberrations may underpin respective translational cross-validation procedures between the two inquiry methods: clinical assessment and functional MRI, as presented in Figure 2.

## Discussion

From the perspective of translational cross-validation, there are numerous important factors to consider.

The first consideration is the involvement of the non-specific task-positive (effort mode) network (43).

In 2015, Kenneth Hugdahl assumed that there exists a task-non-specific effort-mode network in the brain with fronto-temporo-parietal distribution, including inferior and middle frontal gyrus, inferior temporal gyrus, supplementary motor area, and inferior parietal lobule. However, later, Hugdahl states that this extrinsic-mode network is modulated by the nature of the task, namely the extent of the cognitive effort invested (44–46). This hypothesis is further expanded to analyze the dynamic down-regulation of EMN and upregulation of particular regions of the default mode network, such as precuneus, under varying levels of cognitive effort in different psychiatric conditions. As described in the previous sections, the authors do not comment on affective and social-affective tasks, such as Rorschach and TAT. Therefore, their conclusion is limited to cognitive tasks/tests as related, incorporated in fMRI paradigms. In that regard, our results with the depression scale may indicate task-specific modulation of the effort-mode network in terms of up-regulation. This upregulation may well-be explained by the elevated effort invested in the task by the depressed patients. For the paranoid scale, our results may reflect task-specific upregulation of the default mode network in a more complex system of interactions with the salience network that, in effect, leads to aberrant or proximal salience, which can explain most of the positive symptoms in patients with schizophrenia (32, 33). In both measures, the reported effects may be interpreted either as diagnostic-test-specific activations or as modulation effects.

The second consideration comes from the critical paper by the Elliott et al. (47) on the poor test-retest reliability of the common fMRI tasks. In that meta-analysis, pre-defined regions of interest (ROIs), based on an *a-priori* hypothesis, are regarded as a potential caveat, which may determine stringent results, but with poor replicability and limited generalizability. It is evident from this meta-analysis that no particular localization but distributed network interactions are implicated in the mechanisms of mental disorders. Therefore, whole-brain signatures of disease as an alternative to ROI-reporting are proposed as future directions. (47). We have adopted in our studies precisely a whole-brain reporting approach.

The third consideration, also in line with the same publication, is a movement away from pure “experimental” to rather more *naturalistic* stimuli and tests with established psychometric utility (47). Currently, use is specifically designed for experimental control “stimuli,” which are far from real-life and clinical settings and contexts. They do not provide any insights or interpretations beyond experimental settings. Therefore, it is difficult to translate them into clinical utility. Instead, we suggest adopting more robust psychometrical tools in fMRI research under the explicit aspiration of trans-disciplinary validation. In this perspective, clinical self-evaluation tools are assumed as proximal measures of diagnostic task-specific whole-brain functional MRI fingerprints/biological signatures of the clinical condition (state, syndrome), where the two methods cross-validate each other.

In fact, psychological tests and fMRI are noisy measures that are not quantifiable on a large scale, with extensive inter-individual and intra-individual variations. There is no commonly defined “gold standard,” no orientation on how you decide which measures are more valid and more realizable in a case when the two happen to be discrepant. By intuition, one may believe that brain imaging measures are more “objective” and thereby more reliable, but they are as biased as the presumably “subjective” psychological rating scales. Therefore, only the intersection or concordance of the two measures may be regarded as possible evidence for their convergent validity.

Besides, social disability and quality of life outcome measures may be considered possible approaches for external validation of clinical evaluation.

A hybrid design is recommended to combine characteristics of clinically relevant scales and tasks tailored to measure meaningful responses in the brain signals with psychological scale items relevant to the phenomenological core of the syndrome repeated from various perspectives to enhance the BOLD signal. This approach resembles the application of a contrast agent to enhance the image in angiography in interventional medicine. At the next level, the multi-scale variables, including several others, e.g., molecular pathway measures, should be integrated to construct a nomothetic network in psychiatry (36).

## Conclusion and future directions

Our studies may have identified common diagnostic *task-specific* denominators in terms of activations and network modulation at the level of fMRI. However, those common denominators need further investigation to determine whether they signify *disease or syndrome-specific features (signatures)*, which, at the end of the day, raises one more question about the poverty of current conventional psychiatric classification criteria (6). In this study, we propose a novel algorithm for translational validation based on our explorative findings.

**Figure 3:**
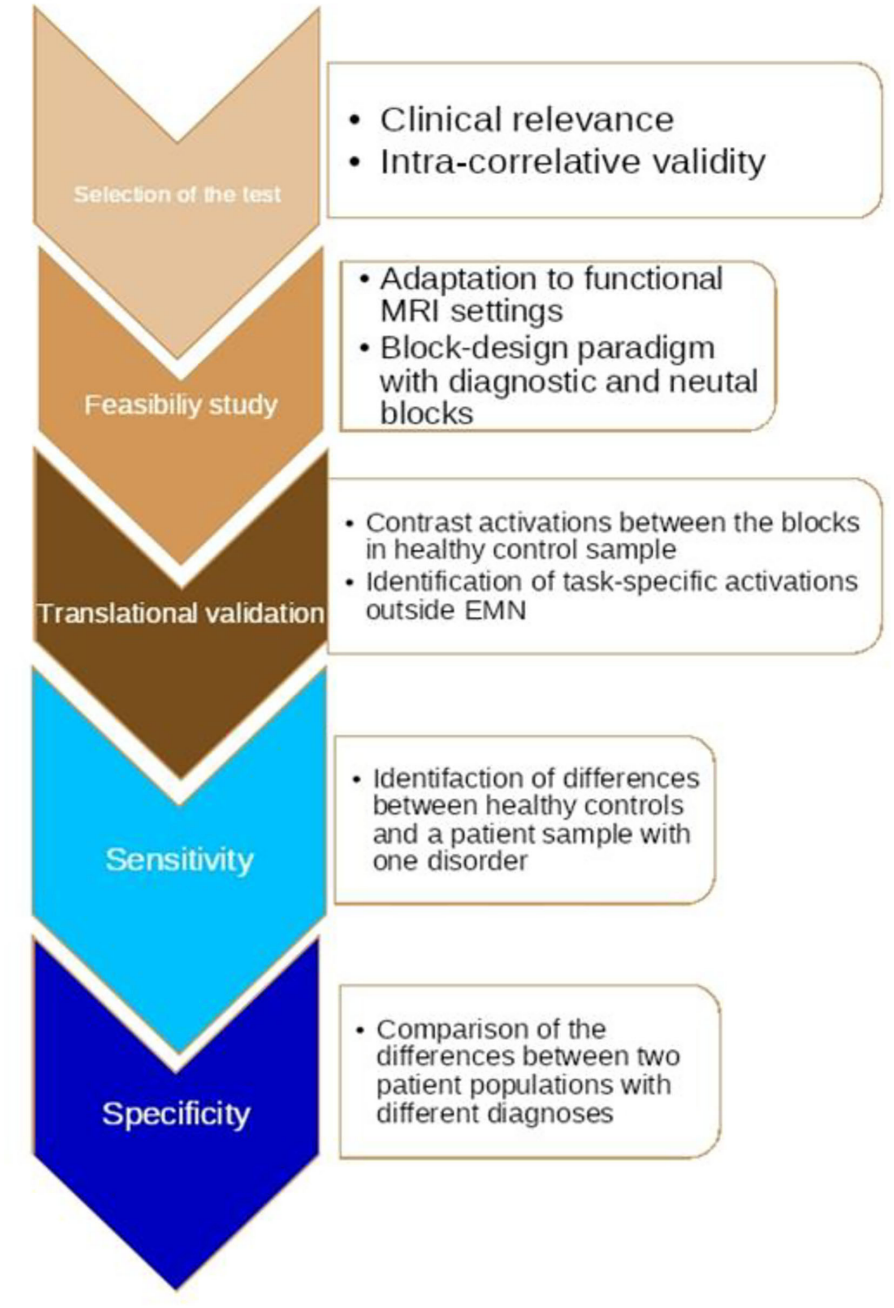
The algorithm of translational cross-validation.

The algorithm includes five steps, as described in more detail in Figure 3: The first one is the pre-selection of a test based on its psychometric characteristics; this suggests that the tool needs to be valid in terms of its ability to distinguish groups by assessment of relevant states or traits in clinical reality. The next step is the adaptation of the test items to a functional MRI paradigm with standardized digital software (e.g., E: Prime). What is critical at this stage is the construction of contrasting blocks of repeated diagnostic stimuli, which may generate a strong enough BOLD third signal; this step includes exploration of the correlation with the diagnostic item responses (scales) of whole brain neural activations in healthy controls as compared to a patient population with a certain diagnosis. The final step is an investigation of the differences between two or more diagnostic classes.

If we manage to translate a sufficient number of tests from psychometrics to functional MRI, this may provide a potentially powerful toolkit for more reliable diagnosis, prognosis, and management of neuropsychiatric disorders, especially if incorporated into broader nomothetic networks, as mentioned above. Further test-retest independent cross-validation needs to follow these explorative findings.

## Data availability statement

The original contributions presented in the study are included in the article/supplementary material, further inquiries can be directed to the corresponding author/s.

## Author contributions

The author confirms being the sole contributor of this work and has approved it for publication.

## Conflict of interest

The author declares that the research was conducted in the absence of any commercial or financial relationships that could be construed as a potential conflict of interest.

## Publisher's note

All claims expressed in this article are solely those of the authors and do not necessarily represent those of their affiliated organizations, or those of the publisher, the editors and the reviewers. Any product that may be evaluated in this article, or claim that may be made by its manufacturer, is not guaranteed or endorsed by the publisher.
